# Highly Tissue Specific Expression of *Sphinx* Supports Its Male Courtship Related Role in *Drosophila melanogaster*

**DOI:** 10.1371/journal.pone.0018853

**Published:** 2011-04-26

**Authors:** Ying Chen, Hongzheng Dai, Sidi Chen, Luoying Zhang, Manyuan Long

**Affiliations:** 1 Committee on Genetics, University of Chicago, Chicago, Illinois, United States of America; 2 Department of Ecology and Evolution, University of Chicago, Chicago, Illinois, United States of America; 3 Department of Neurobiology and Physiology, Northwestern University, Evanston, Illinois, United States of America; University of Dayton, United States of America

## Abstract

*Sphinx* is a lineage-specific non-coding RNA gene involved in regulating courtship behavior in *Drosophila melanogaster*. The 5′ flanking region of the gene is conserved across *Drosophila* species, with the proximal 300 bp being conserved out to *D. virilis* and a further 600 bp region being conserved amongst the *melanogaster* subgroup (*D. melanogaster, D. simulans, D. sechellia, D. yakuba, and D. erecta*). Using a green fluorescence protein transformation system, we demonstrated that a 253 bp region of the highly conserved segment was sufficient to drive *sphinx* expression in male accessory gland. GFP signals were also observed in brain, wing hairs and leg bristles. An additional ∼800 bp upstream region was able to enhance expression specifically in proboscis, suggesting the existence of enhancer elements. Using anti-GFP staining, we identified putative *sphinx* expression signal in the brain antennal lobe and inner antennocerebral tract, suggesting that *sphinx* might be involved in olfactory neuron mediated regulation of male courtship behavior. Whole genome expression profiling of the *sphinx* knockout mutation identified significant up-regulated gene categories related to accessory gland protein function and odor perception, suggesting *sphinx* might be a negative regulator of its target genes.

## Introduction

The genetic basis of species-specific courtship behavior has been one of the major interests of evolutionary biology. Behavioral differences between *Drosophila* species have been well-documented, especially the ones that influence mate choices and have important fitness consequences [1]. But little study has been done to reveal whether there is any relationship between these phenotypic differences and lineage specific genes. Our recent study of *sphinx* has been one of the few studies that directly related novel behavior to newly evolved gene. *Sphinx* is a lineage specific chimeric gene [2], [3] involved in regulating male courtship behavior [4]. The *sphinx* gene was formed by the insertion of a retroposed sequence of the ATP synthase F-chain gene (CG4692) from chromosome 2 into the 102F region of chromosome 4 (first exon), recruiting sequences upstream to form an intron and a second exon [2]. The sphinx gene appears to be functional because the gene contains indel polymorphisms only in the non-exonic sequences; it has a rate of evolution significantly above neutral expectations, suggesting rapid adaptive evolution. However, although it is derived, in part, from a protein-coding gene, it is most likely a noncoding RNA (ncRNA) because its parental-inherited coding regions are disrupted by several nonsense mutations [2].

We previously showed that knocking-out of this gene led to increased male-male courtship in *D. melanogaster*, while leaving other aspects of mating behavior unchanged [4]. Comparative studies of courtship behavior in other closely related *Drosophila* species suggested that this mutant phenotype of male-male courtship was the ancestral condition, since these related species showed much higher levels of male-male courtship than *D. melanogaster*. The recruitment of *sphinx* in *D. melanogaster* therefore, might have increased male-female mating by suppressing male-male courtship behavior [4].

Male courtship in *Drosophila* is an elaborate ritual involving multiple sensory inputs with olfactory and/or gustatory stimuli being particularly important during mate recognition [5], [6]. In flies, different subsets of olfactory receptor neurons (ORNs) of the olfactory appendage, the antenna and the maxillary palps project axons to different functional processing units called glomeruli in the antennal lobe (AL). The AL is the primary olfactory association center in insects where ORNs synapse onto second order neurons called projection neurons (PNs). An essentially complete olfactory map has been constructed by large-scale genetic efforts to label ORNs expressing each of the 62 known OR genes and map their projections to approximately 50 morphologically defined glomeruli in the adult AL [7], [8]. The axons of PNs project to the mushroom body (MB) and lateral horn via inner antennocerebral tract (iACT) [9], [10]. The organization of gustatory system is more dispersed than olfactory system. The main taste organs are the labial palps at the distal end of the proboscis, and the labral and cibarial sense organ inside the pharynx [11]. Gustatory receptor neurons in these sensilla project axons to the subesophageal ganglion(SOG) of the brain [12].

Here, we utilized a promoter GFP transformation system to dissect promoter region of *sphinx* and to investigate its expression pattern in relation to its function. We found that 1 kb upstream region of *sphinx* was able to drive GFP expression in accessory gland, and possibly peripheral and central nervous system, suggesting existence of putative promoter elements within this region. The highly tissue specific expression pattern also supported *sphinx*'s reproductive related role. We carried out microarray analysis of a *sphinx* mutant to identify possible pathways in which *sphinx* might be involved. Results from our analysis suggest *sphinx* might function as a negative regulator in the courtship network.

## Results and Discussion

### Promoter region of *sphinx*

**Figure 1:**
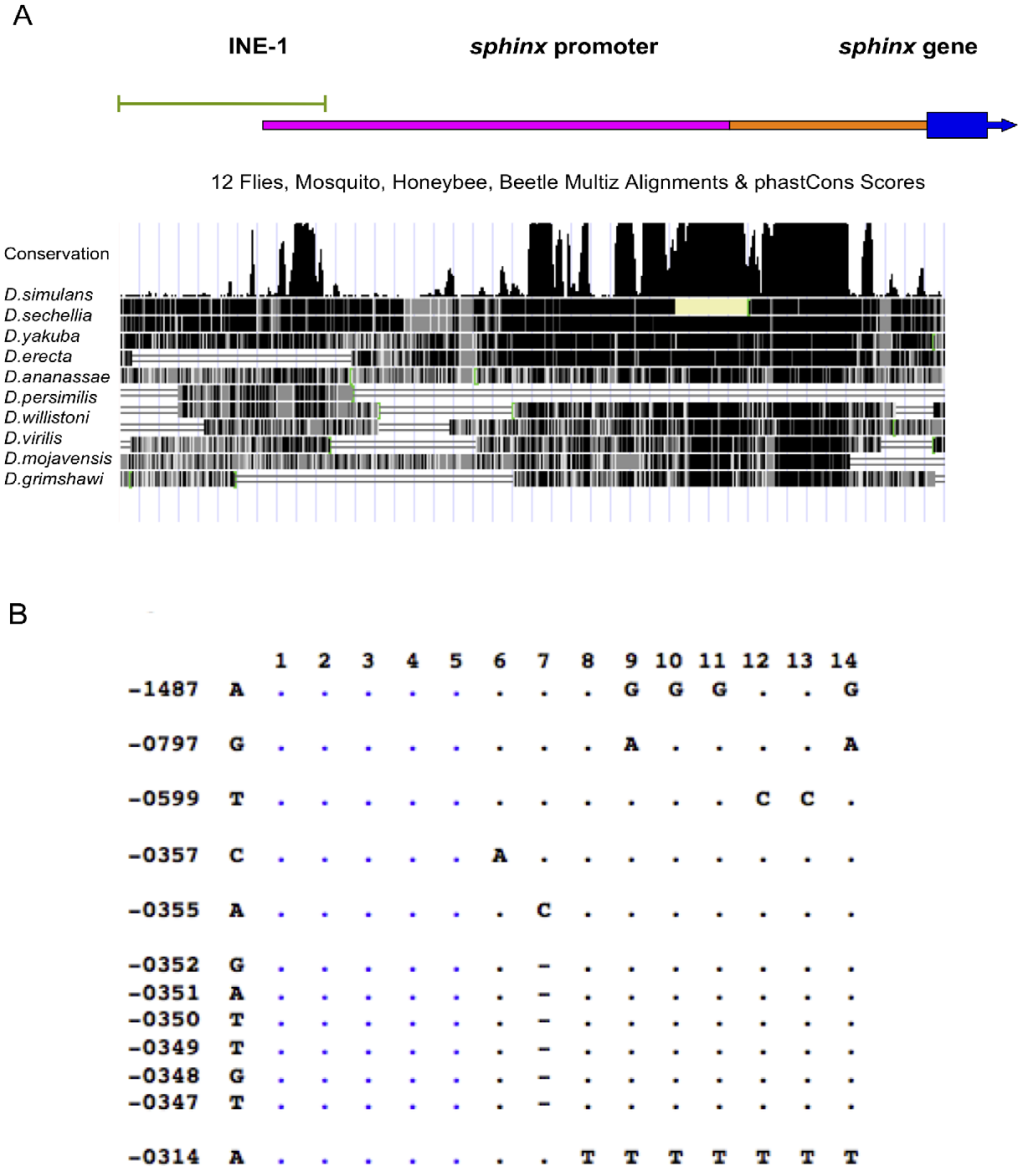
Sequence conservation of *sphinx* promoter region. (A) Multiple species alignment of the promoter region of *sphinx.* Top panel indicates relative position of INE-1 element (green line), 265 bp region (orange bar) used in GFP transformation and additional 802 bp (pink bar) for 1067bp construct. Lower panel: sequence conservation of promoter region, black shade indicates conserved region. (B). Segregating sites in *D. melanogaster*. The nucleotide positions from the transcription start site are indicated in the first column. The second column shows the consensus nucleotides. The dots indicate that the nucleotides are identical to those of the consensus. The blue color represents the common haplotype. The first row contains the line numbers that were sequenced: 1. TWN.4 2. Yep2 3. Yep25 4. Ok17 5. HG84 6. Yep10 7. NFS 8. La79 9. TWN30 10. TWN35 11. TWN38 12. ZS30 13. ZS56 14. TWN 27.

We identified several conserved elements in the 5′ regulatory region of *sphinx* from multiple species syntenic alignment [4]. The very proximal conserved region encompassed ∼300 bp upstream of the transcriptional start site and is very conserved out to *Drosophila virilis* (90% identical). The homology extends further upstream to around −600 bp position with high sequence conservation within the *melanogaster* subgroup. An additional distal conserved element sits around −850 to −1000 bp region (Figure 1A).

To further investigate the evolutionary processes in the *sphinx* promoter region, we performed a polymorphism survey among world-wide populations. Consistent with the divergence data, the polymorphism level of the promoter region in *D. melanogaster* was very low. We sequenced 12 lines from a geographically diverse panel of populations and found only 7 segregating sites among the 1.3 kb region (Figure 1B). The average nucleotide diversity (π) was estimated to be 0.00163, which was about half the level of diversity seen in the 4^th^ chromosome CG11091-toy region (0.0028), sequenced in the same lines. Thus the promoter region of *sphinx* seems to be under selective constraint.

### Expression patterns of *sphinx*

To dissect the promoter region and understand the molecular functions of *sphinx*, we used Green Fluorescent Protein (GFP) as a reporter [13], [14] to investigate the expression pattern of *sphinx* in various tissues. We generated two P-element derived constructs of P*sphinx*-GFP that contained GFP tagged 265 bp sequence of the proximal conserved region and 1067 bp (which includes all three conserved regions) of the 5′ upstream genomic region of *sphinx*. By the standard p-element transformation procedure, we obtained 12 transgenic lines with a 265 bp fragment insertion and 6 lines with a 1067 bp insertion on different chromosomes. We examined GFP signals of all the transgenic lines at different developmental stages: embryo, three larvae stages, pupae (Figure S1), and in a variety of tissues: head, wing, leg, testis, ovary, accessory gland, and brain (Figure S2, S3). Consistent patterns of GFP expression were observed in the brain, accessory gland, wing and leg across all transgenic lines, with noted differences of expression in proboscis between the short and long promoter-GFP constructs (see below).

**Figure 2:**
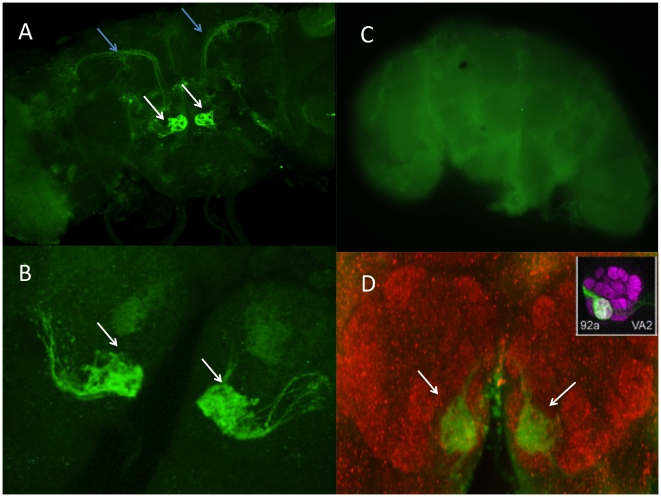
P*sphinx*-GFP line was stained with anti-GFP. (A) antennal lobe (white arrowhead) and inner antennoglomerular tract (blue arrowhead); (B) zoomed-in image of the two glomeruli. (C) negative control for immunostaining. (D) Double-staining with anti-GFP to visualize *sphinx* expression in the glomerulus VA2 (green), and the synaptic marker mAb nc82 (red) to visualize the glomerular structure of the antennal lobe.

**Figure 3:**
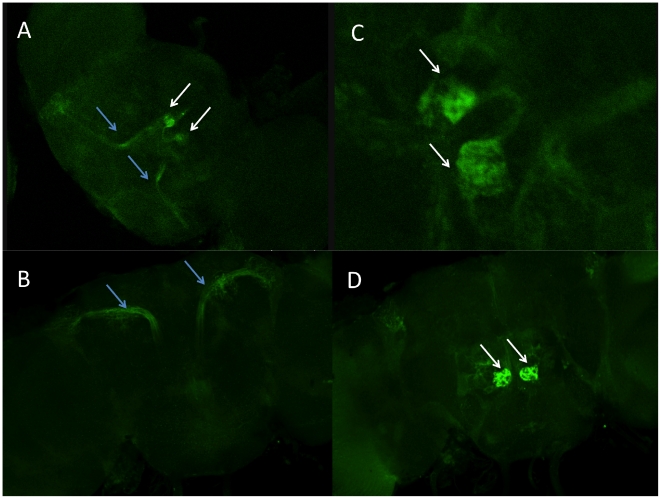
Male (A and C) and Female brain (B and D) GFP signal. Glomeruli and inner antennoglomerular tract were indicated by white and blue arrowhead, respectively.

Anti GFP staining in the *Drosophila* brain showed a distinctive GFP signal in a pair of glomeruli of the antennal lobes (Figure 2). At a slightly different confocal plane, we observed signals in the inner antennal glomerular tract (Figure 2A). To further identify the exact glomeruli in which GFP signal was visualized, we counter stained the brains of P*sphinx*_GFP lines with neurophile marker nc-82 (Figure 2D). By comparing to the 3D reconstruction of the antennal lobe [15], we observed that the structure and positioning of GFP stained glomerulie is very similar to glomeruli VA2, which corresponds to Or92a projection in the AL. Odor ligands that activate Or92a and its corresponding glomerulus are carvone and octanal. The neuronal expression pattern of *sphinx*, however, did not appear to be sexually dimorphic, as there were little observable differences between male and female brains (Figure 3).

The possible co-expression of *sphinx* and *Or92a* in VA2 suggests that *sphinx* might function in the same pathway as *Or92a*. *Or92a* has not previously been identified in courtship behavior, but it is one of the few OR genes that were found to be co-expressed with GR genes, in this case with *Gr21a* in ab1 of large basiconics of antenna [7]. Our microarray analysis (details see below) suggested that both *Or92a* and *Gr21a* genes were significantly up-regulated in the *sphinx* mutant with p-value of 0.014 and 0.017 respectively. Thus *sphinx* might play an integrative role between the olfactory and gustatory system.

**Figure 4:**
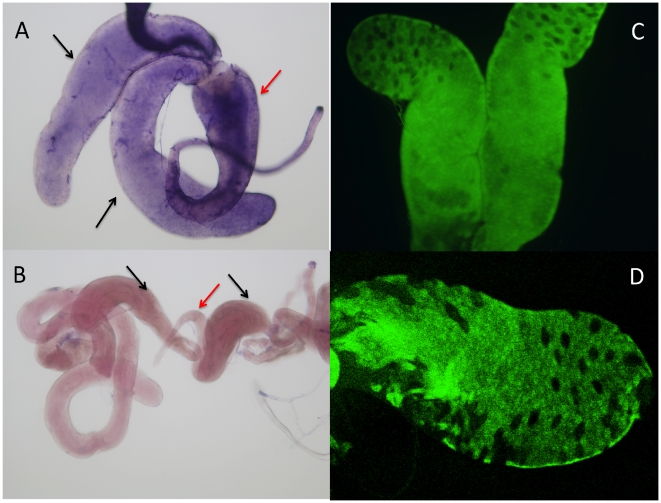
*sphinx* expression in accessory gland. (A) RNA in situ hybridization showing *sphinx* expression in accessory gland (black arrowhead) and ejaculation duct (red arrowhead); (B) negative control for RNA is situ; (C) GFP signal of accessory gland visualized under fluorescence microscope; (D) GFP image under confocal microscope.

A strong and consistent signal was observed in male accessory glands that are known to be involved in regulating male reproduction and courtship behavior [16] (Figure 4). There are two types of secretory cells: main cells and secondary cells. Only main-cell secretions are essential for the short-term inhibition of remating [17]. In the accessory gland, it was the main cells but not the secondary cells that show strong GFP expression (Figure 4C, D). There was, however, no GFP expression in male testis and female ovaries. The endogenous expression of *sphinx* in male accessory gland was confirmed by antisense RNA *in situ* hybridization, which clearly showed that *sphinx* mRNA exists in the accessory gland and anterior ejaculation duct (Figure 4A). We found very little or no *sphinx* mRNA in the testes and ovaries. This is consistent with the GFP expression pattern. The lack of GFP signal in ejaculation duct was probably due to fusion of GFP protein with target gene product making it difficult to be secreted from the accessory gland to the anterior ejaculation duct.

**Figure 5:**
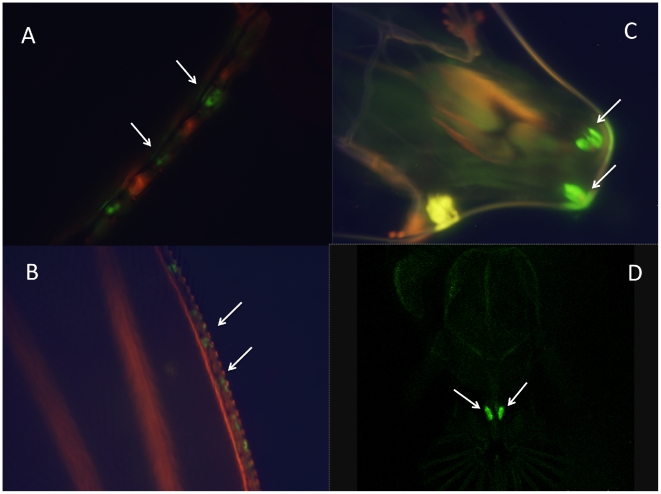
*sphinx* expression in peripheral nervous system. (A) leg bristle; (B) wing bristle; (C) larval anterior spiracles; (D) adult proboscis (labral sensory organ). C and D are specific for 1067 bp insertion.

**Figure 6:**
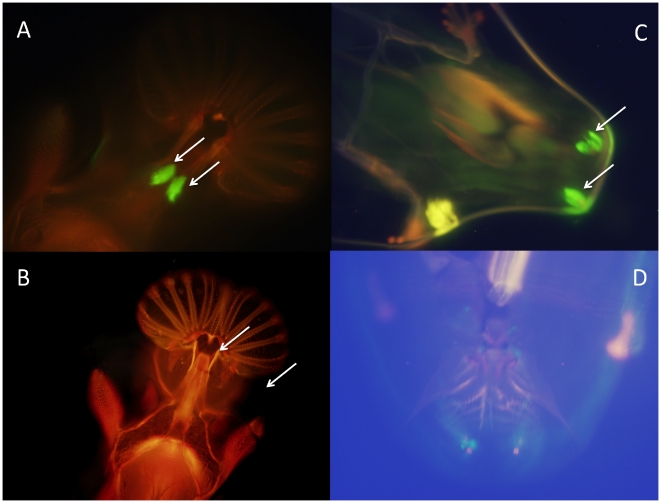
The differences between 1067 bp and 265 bp GFP transformation lines. *sphinx* expression in adult proboscis of 1067 bp (A) and 265 bp (B) insertions, and larval terminal organs of 1067 bp (C) and 265 bp (D) insertions.

Furthermore, we observed GFP expression in chemosensory organs in both male and female adults: the bases of bristles on forelegs (Figure 5A); and the bristles of the anterior margin of the wings (Figure 5B) in all the lines. We also observed expression in the labral sense organ in the adult proboscis and the corresponding larval and pupae terminal sensory organ in the lines containing the long promoter insert (Figure 5C, D, 6A, C) but no expression in the lines containing the shorter construct (Figure 6B, D); Chemosensory organs are important in *Drosophila* pheromonal communication, especially in male perception of cuticular hydrocarbons during courtship [18], [19].

The 12 lines with the 265 bp insertion show very similar pattern of expression with the 6 lines with longer insertion. The only difference exists in the labral sense organ in the proboscis where we see clear expression in the 6 GFP transformation lines carrying 1076 bp insertion (Figure 5A, C, Figure 6A, C), but little or no signal in the lines carrying only the 265 bp promoter region (Figure 6B). There was also no evidence of expression in larvae terminal sensory organ with the 265 bp insertion lines (Figure 6D). These results implied that the promoter region of *sphinx* might have different functional units, with upstream 800bp carrying enhancer element that might be important for *sphinx*'s expression in labral sense organ.

We were able to confirm *sphinx* expression in accessory gland by in situ hybridization and RT-PCR. We do not have independent evidence supporting the expression of the *sphinx*-promoter driven GFP expression in pheripheral and central nervous system due to technical difficulties. Yet previous behavior experiment and the consistent GFP expression patterns among replicate lines strongly suggest that the observed signals are real.

### Whole genome expression profiling of the *sphinx* mutant

The expression of *sphinx* in accessory gland indicates *sphinx*'s involvement in the male reproductive system, while the possible expression in chemosensory organs and AL suggests its possible neuronal function, suggesting the behavior of the *sphinx* mutant might involve expression changes in many other genes. Using Affymetrix microarrays, we compared expression profiles of the *sphinx* null mutant versus *Oregon-R* (mutant's genetic background) flies to search for candidate genes that might interact with *sphinx*. We identified differentially expressed genes as those that showed at least a 2-fold (up or down) difference in expression, with a *p* value of <0.001. This resulted in a list of 84 up-regulated (Table S1) and 55 down-regulated genes (Table S2) in the *sphinx* mutant compared to *Oregon-R*. *Obp99d* was significantly up-regulated in the *sphinx* mutant (P<<0.001), which was probably not by accident as it was previously showed that the transcript abundance of this gene affected mating speed [20]. *Obp56h* and *Elav* were significantly down-regulated in the *sphinx* mutant, which might also potentially interact with sphinx. It was rather surprising that two female spermatogenesis genes (*kelch* and *E2F*) were among the down-reulated gene list. It might be that male and female sometime share the use of same genes during gametogenesis, or that sphinx might affect female reproductive behavior in aspects that have not been uncovered.

To further identify potentially important biochemical processes in a statistically rigorous way, we made use of the freely available software package CATMAP (http://bioinfo.thep.lu.se/Catmap) [21]. This program assigns significance to gene categories based on their relative statistical ranking or representation within the data set. We ran Catmap analysis on the ranked gene list based on the Bayes t statistic of all genes for over-representation of functional categories from a number of biological databases, including GO and Interpro, and several customized databases that contain microarray data and functional classifications from previously published studies. The top lists of over-represented up-regulated gene categories in the *sphinx* mutant line are accessory gland protein and chemosensory and odorant receptor genes, while down regulated genes show no bias towards gene categories related to male reproduction or courtship (Table 1). One plausible explanation is that *sphinx* acts as a negative regulator in the biological processes related to accessory gland protein and neuropeptide secretion. Thus the inability of *sphinx* mutants to discriminate male from female might due to the fact that certain sensory circuits, which are normally only turned on in the presence of females, are being turned on constitutively.

**Table 1:** Process level comparison between *sphinx* mutant and wild type.

Categories	p value
Up-regulated Gene Categories	
Accessory gland protein	8.19E-25
Serine proteases and their homologs	6.43E-09
BP_GO:0045297 post-mating behavior	4.51E-08
Chemoreceptor gene family	1.93E-07
MF_GO:0004295 trypsin activity	2.55E-06
MF_GO:0004984 olfactory receptor activityMF_GO:0005549 odorant binding	5.63E-061.21E-04
Down-regulated Gene Categories	
CC_GO:0033279 ribosomal subunit	5.32E-21
BP_GO:0007242 intracellular signaling cascade	5.13E-12
CC_GO:0005838 proteasome regulatory particle	1.70E-10
Box C/D and box H/ACA families of snoRNA genes	1.83E-10
Steroid- and radiation-triggered programmed cell death	1.45E-09
Pathways regulating cell size and cell-cycle progression	1.91E-09
BP_GO:0006725 aromatic compound metabolic process	1.68E-07
BP_GO:0007243 protein kinase cascade	1.92E-07

Significantly up- or down-regulated functional categories at false discovery rate <0.01, with significance determined using Catmap. BP_GO: biological process gene ontology; MF: molecular function; CC: cellular component.

## Materials and Methods

### Fly Strains

All fly strains, w1118 and GFP transformant lines, were kept at 25c on standard agar medium.

### Sequence comparison

Homologous sequences of the *sphinx* gene and its upstream region were retrieved by running BLAST against the *D. yakuba* and *D. simulans* genome assemblies (http://flybase.bio.indiana.edu/blast/). Syntenic alignment files were downloaded from UCSD genome browser (http://genome.ucsc.edu).Polymorphism data of *D. melanogaster* 5′ regulator region were collected from a worldwide collection of lines: OK17, HG84, Z(s)56, Z(s)30 from Africa; yep2, yep10, yep25 from Australia; 253.4, 253.27, 253.30, 253.30 and 253.38 from Taiwan. Primers used to amplify 5′ regulatory region were 5′ CCCTGGAGACCATTTCGTTA 3′ and 5′ TCCGCACATTTCATTTTCAA 3′. PCR products were sequenced directly after purification [Qiagen (Valencia, CA) kit] on an ABI automatic DNA sequencer (Applied Biosystems, Foster City, CA) using DyeDeoxy terminator reagents.

### GFP transformation

265 and 1067 base pair fragments from the upstream region of *sphinx* were amplified by PCR and cloned into the pTOPO vector. The primer sequence pairs are as follows:

UP*SPHINX*-3PRIM2: GATAAGTTTTCCCGGCCGCTTTA (GATAAGTTTTCGCTATCGCTTTA) (Xma 1)

UP*SPHINX*-5′1-4: CTGCAGGGCAACATCAGA::

(GCGCGTGGCAACATCAGA) (Pst 1)

UP*SPHINX*-5′4: GGGCGGGCAAACTTTACAA.

After digestion by proper restriction enzymes, these fragments were inserted into a GFP expression vector pEGFP-N1. The chimeric p*Sphinx*-GFP fragments were introduced in a P-element vector pCasper4. Microinjection was performed on w1118 (white eye) embryos. Successful transformants (red eyes) were screened from progenies of the crosses between the hatched injected flies to w1118 individuals. Red eye offspring were further inbred and purified for several generations until homozygous lines were established. GFP expression was visually examined using UV illumination with an Olympus BX60 stereomicroscope and fluorescence module.

### Anti-GFP staining

Dissection and antibody staining of adult brain whole mounts was performed exactly as described in [15], using the nc82 antibody (kindly provided by Professor Reini Stocker), which was visualized with a 1∶100 dilution of goat anti-mouse IgG coupled to CY3 (Jackson ImmunoResearch). Expression of *Psphinx*-GFP was visualized with a 1∶1000 dilution of anti-GFP antibody (Molecular Probes) and a 1∶250 dilution of goat anti-mouse secondary antibody coupled to Alexa Fluor 488 (Molecular Probes). Brains were mounted in Vectashield (Vector Labs) using small cover slips as spacers and analyzed with a LeicaSP5 2photon confocal microscope.

### Oligonucleotide Microarray Analysis

Changes in transcript abundance were measured using *D. melanogaster* whole genome oligonucleotide microarrays 2.0 (Affymetrix). Total RNA was extracted from 5 days old adult male flies of oregon R and *sphinx* mutant *sphinx*720RW [4] by using Qiagen Rneasy mini kit according to the manufacturer's procedures. We performed three biological replicates of each genotype. All Affymetrix protocols were performed at the University of Chicago Functional Genomics Core Facility. The cRNA probe was generated by using standard Affymetrix protocols (www.affymetrix.com). Fragmented biotinylated probe was then hybridized to *D. melanogaster* whole genome arrays. Washing, labeling (streptavidin-phycoerythrin), and scanning followed standard procedures at the Core Facility.

### Statistical Analysis

To calculate gene expression measures, the data sets were normalized as follows. Raw image files were converted to probe set data (.cel files) in Microarray Suite (MAS 5.0). The 20 probe set data files were normalized together, and expression values were determined, using the Robust Multichip Average method [22], implemented in the Affymetrix package (version 1.4.14) of the free statistical programming language R (www.r-project.org). We calculated a new *t* test value for all gene changes, using a more stringent two-tailed Student's *t* test and assuming unequal variance. We selected probes that showed at least a 2-fold (up or down) difference in expression and had a *p* value of <0.001. This resulted in a list of 139 differentially expressed genes (84 up-regulated, 55 down-regulated).

For Catmap analysis, a ranked gene list based on the Bayes *t* statistic from the Goldenspike [23] analysis was used as input. The Wilcoxon rank sum was used to generate a score based on the sum of the rankings of all genes with a particular functional annotation, and the significance of that score (the *p* value) was calculated analytically based on a random gene-rank distribution [21]. Gene categories were considered significantly differentially regulated at FDR (false discovery rate) <0.01.

## Supporting Information

Figure S1 Representative GFP images at different developmental stages.
(TIF)

Figure S2 Representative GFP images of (A) male head (B) female head (C) male accessory gland (D) female ovary.
(TIF)

Figure S3 Representative GFP images of (A) male foreleg (B) female foreleg (C) male wing (D) female wing.
(TIF)

Table S1 Annotation of 84 up-regulated genes in the *sphinx* mutant compared to *Oregon-R*.
(XLSX)

Table S2 Annotation of 55 down-regulated genes in the *sphinx* mutant compared to *Oregon-R*.
(XLSX)
