# Supplementary figures and images for: Highly Tissue Specific Expression of Sphinx Supports Its Male Courtship Related Role in Drosophila melanogaster
Source: PLoS One. 2011 Apr 26;6(4):e18853. doi: 10.1371/journal.pone.0018853 (PMC3082539; doi:10.1371/journal.pone.0018853)

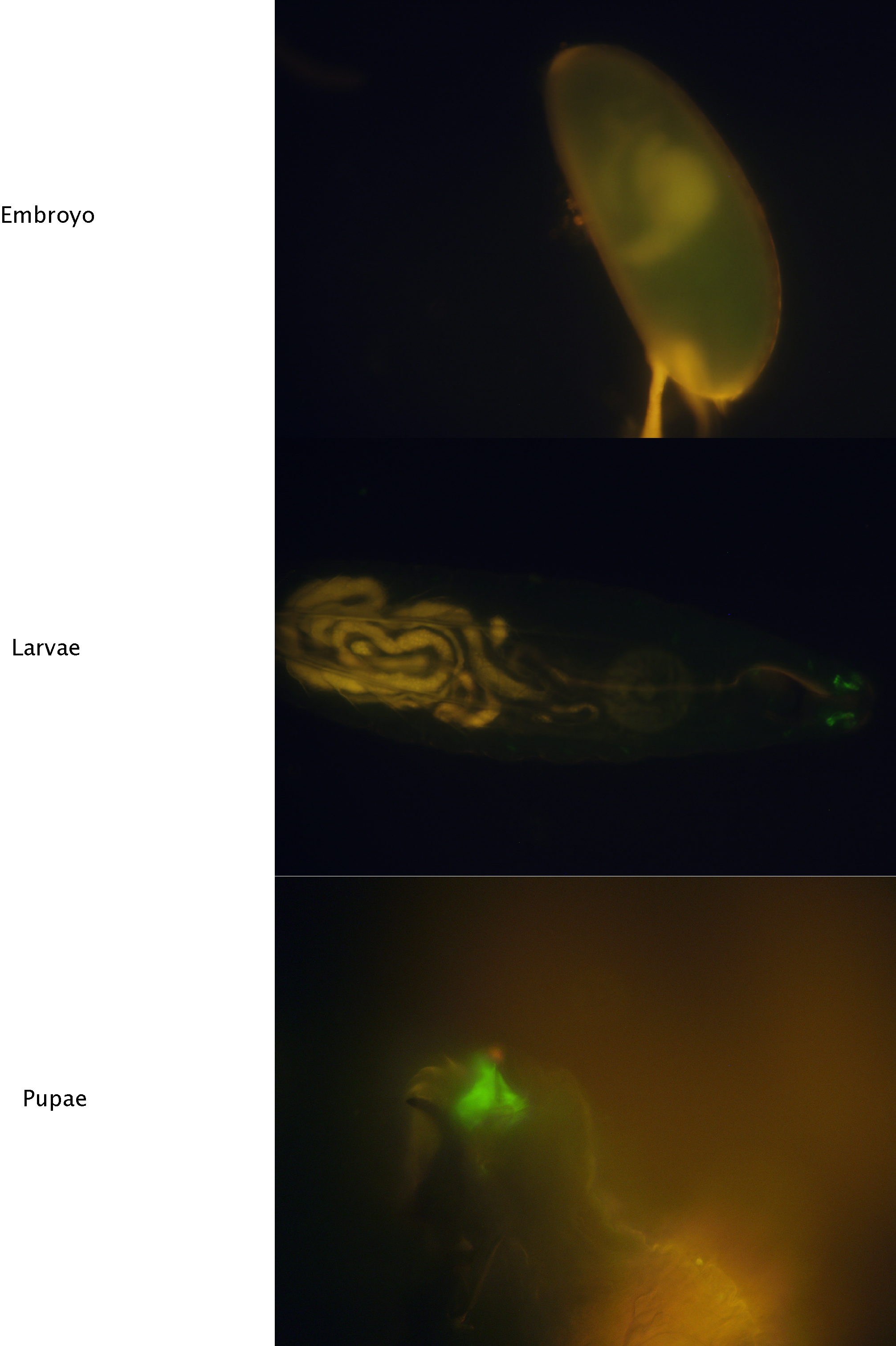

Supplement: Figure S1 — Representative GFP images at different developmental stages. (TIF) [file pone.0018853.s001.tif]

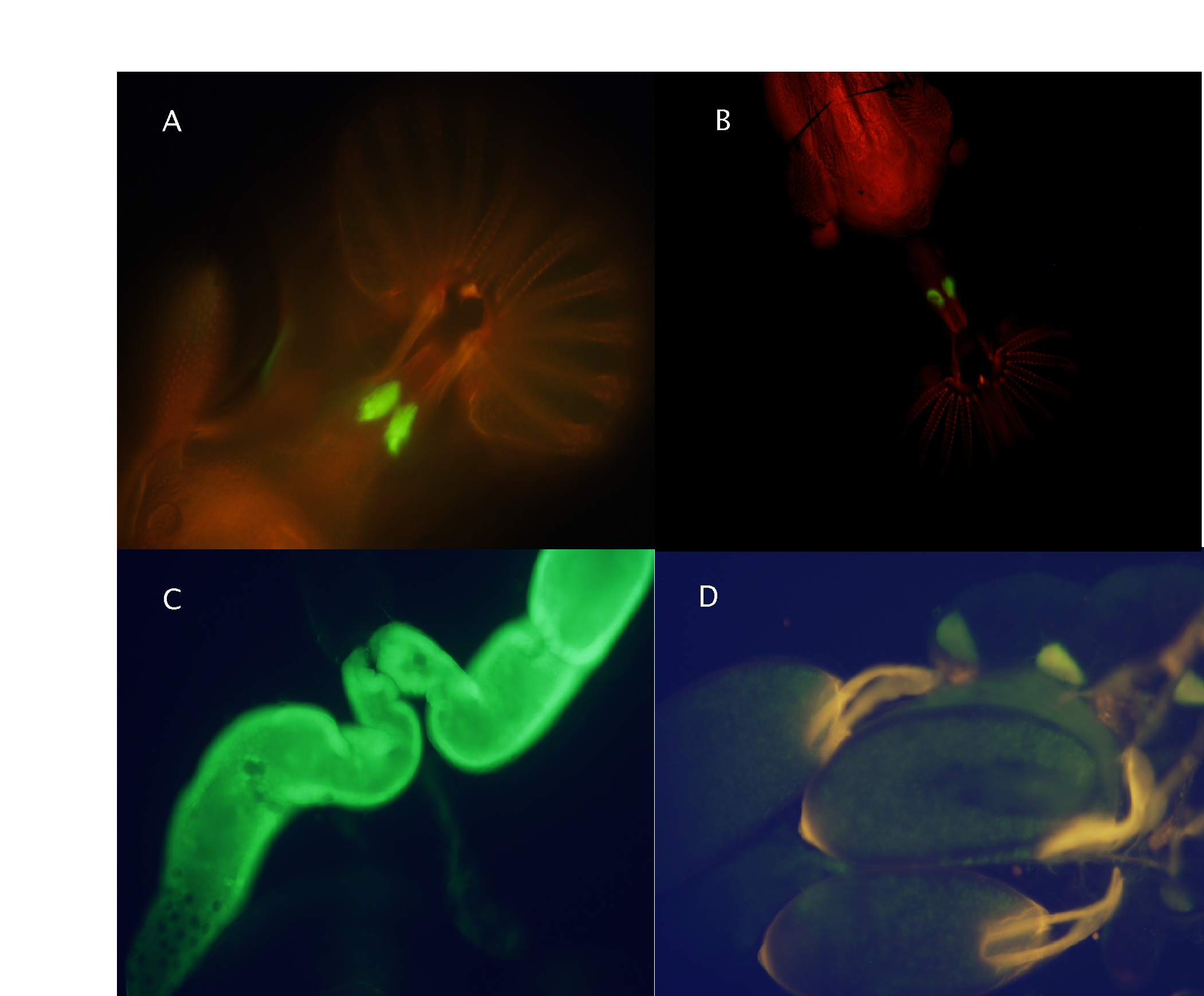

Supplement: Figure S2 — Representative GFP images of (A) male head (B) female head (C) male accessory gland (D) female ovary. (TIF) [file pone.0018853.s002.tif]

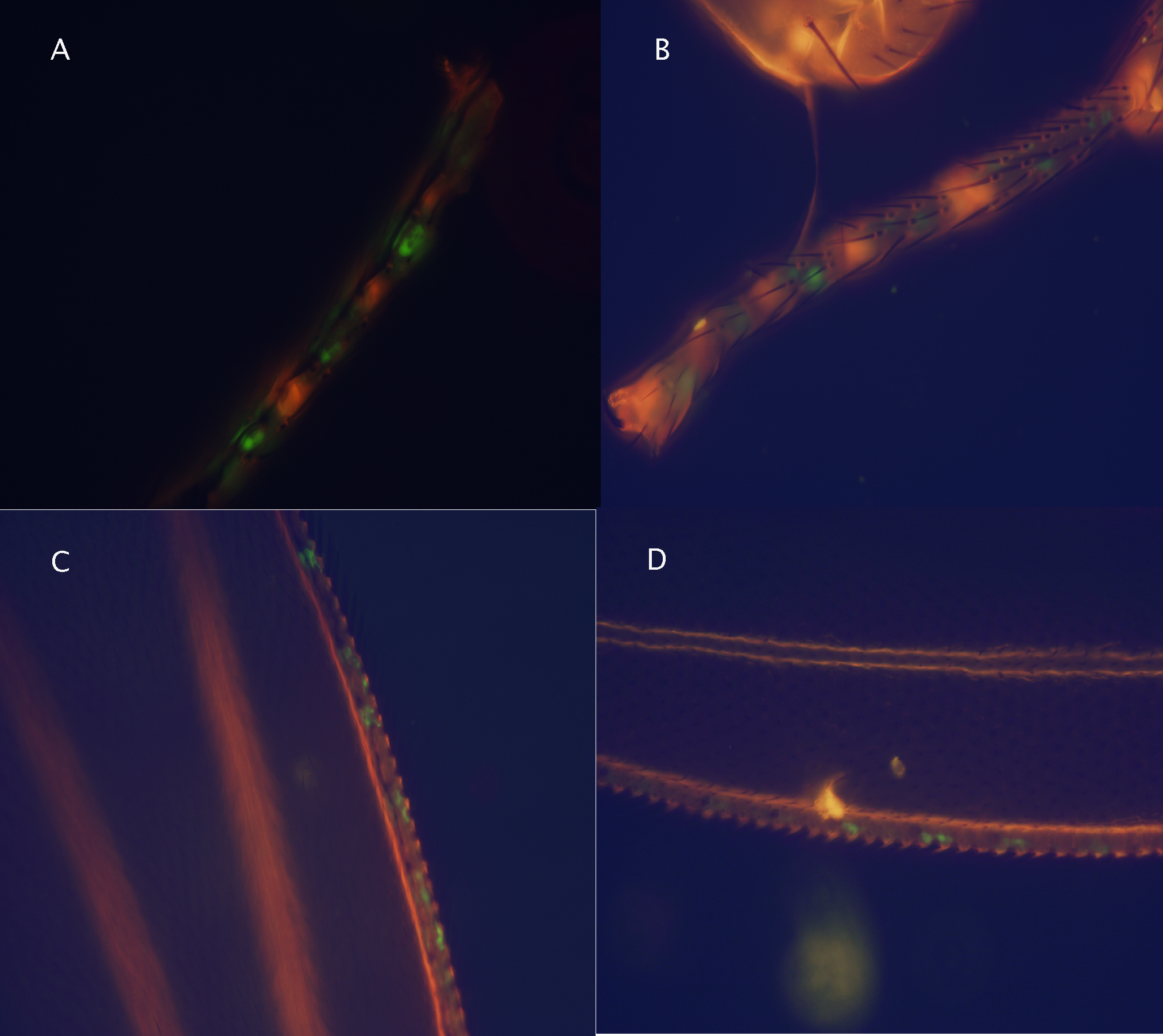

Supplement: Figure S3 — Representative GFP images of (A) male foreleg (B) female foreleg (C) male wing (D) female wing. (TIF) [file pone.0018853.s003.tif]
